# Traumatic Apoplexy

**Published:** 1904-11-12

**Authors:** 


### TRAUMATIC APOPLEXY.

The occurrence of apoplexy after a head injury and independent of laceration of a blood-vessel at the time of the injury is a pathological phenomenon which deserves more attention than it has generally received. There were two inquests held in London recently in each of which the medical evidence, as reported in the newspapers, was to the effect that death was due to cerebral hæmorrhage independent of certain injuries received a short time previously and which it had been alleged had contributed to the fatal issue. It is manifest that the medico-legal considerations involved in such cases are of great moment, and, if for this reason alone, it is desirable to have the pathological features of traumatic apoplexy thoroughly worked out and placed on permanent record. Dr. Pearce Bailey,[1] who has made an excursion into the literature of the subject, mentions three varieties of apoplexy occurring after head injuries, in which the connection between the injury and the apoplexy is close and indisputable—it being assumed that diseased cerebral arteries are necessary precedents to the hæmorrhage, while the extent to which injuries contribute to pathological changes in the vessels is undecided. His classification is as follows:—(1) Apoplexy occurring simultaneously with the injury; (2) apoplexy occurring shortly after the injury; (3) apoplexy occurring a long time after the injury. In cases belonging to the first group—the most important group from a medico-legal standpoint—the patient, immediately after a blow on the head, develops symptoms of hæmorrhage in the brain. The blow may not have been severe and evidence of injury to the scalp or skull may be absent. The symptoms are those of spontaneous apoplexy and may be quickly fatal; or there may be hemiplegia involving arm, leg, and face, with more or less recovery. The explanation of these cases is that a weakness of the cerebral arteries has existed, and that their walls have given way when subjected to a sudden rise in blood pressure. It may be that the increased blood pressure is due to the mechanical effects of the blow, but probably the psychic factors of fright and excitement are the most potent causes.

In cases belonging to the second group the hæmorrhage occurs a few days or weeks after a head injury; and it takes place in the neighbourhood of the fourth ventricle and the aqueduct of Sylvius, and is fatal. Bollinger is of opinion that the hæmorrhage is preceded by softening of the neighbouring brain substance, but this has not been proved. The injury has been invariably to the head. It may or may not cause unconsciousness. In any event the patient recovers for the time, and is able to return to work. After a few days or weeks, headache, somnolence and coma ensue, with paralysis of the extremities or of the cranial nerves, or an apoplectic seizure may occur without warning. Many of the cases belonging to this group have been in young persons.

In the third group the symptoms point to a slow increase of vascular obstruction in parts which have long before been the seat of trauma, and are those of slow thrombosis. The preceding injury in most instances has been severe, and accompanied by fractured skull and laceration of the brain. The increase in symptoms begins after a lapse of many years. Dr. Bailey mentions two cases in which the apoplexy came on 10 and 26 years respectively after the original injury.

[1] Medical Record, Oct. 1, 1904.

